# Characterizing Bilingual Effects on Cognition: The Search for Meaningful Individual Differences

**DOI:** 10.3390/brainsci11010081

**Published:** 2021-01-09

**Authors:** Kristina C. Backer, Heather Bortfeld

**Affiliations:** 1 Department of Cognitive and Information Sciences, University of California, Merced, CA 95343, USA; 2 Department of Psychological Sciences, University of California, Merced, CA 95343, USA

## Abstract

A debate over the past decade has focused on the so-called bilingual advantage—the idea that bilingual and multilingual individuals have enhanced domain-general executive functions, relative to monolinguals, due to competition-induced monitoring of both processing and representation from the task-irrelevant language(s). In this commentary, we consider a recent study by Pot, Keijzer, and de Bot (2018), which focused on the relationship between individual differences in language usage and performance on an executive function task among multilingual older adults. We discuss their approach and findings in light of a more general movement towards embracing complexity in this domain of research, including individuals’ sociocultural context and position in the lifespan. The field increasingly considers interactions between bilingualism/multilingualism and cognition, employing measures of language use well beyond the early dichotomous perspectives on language background. Moreover, new measures of bilingualism and analytical approaches are helping researchers interrogate the complexities of specific processing issues. Indeed, our review of the bilingualism/multilingualism literature confirms the increased appreciation researchers have for the range of factors—beyond whether someone speaks one, two, or more languages—that impact specific cognitive processes. Here, we highlight some of the most salient of these, and incorporate suggestions for a way forward that likewise encompasses neural perspectives on the topic.

## 1. Introduction

Language is a core component of our daily lives, allowing us to connect with others across a wide range of personal, social, and professional contexts. Given the increasingly large proportion of the world’s population that is bilingual or multilingual, efforts to understand the cognitive impacts of language experience have only accelerated in recent years. Correspondingly, appreciation is accruing for the role that sociocultural e.g., [1,2]- and developmental factors e.g., [3,4] play a role in the cognitive impact of speaking more than one language. For example, the degree to which switching from one language to another occurs, and when it occurs, varies enormously across both individuals and groups. Consequently, there is great interest in understanding how one’s experience using two or more languages, and doing so at different stages of life, modulate other aspects of cognitive processing. In particular, an initial focus on whether speaking more than one language confers a domain-general advantage for bilinguals relative to monolinguals in executive control—the so-called “bilingual advantage”—in recent years has shifted to an approach better characterized as looking for “bilingual effects.” This approach incorporates nuanced characterization of language usage across the lifespan in bilingual/multilingual individuals and the context in which their language(s) developed and are used for an overview, see [5].

We’ll first summarize the debate about the cognitive impact of bilingualism/multilingualism. A general perspective has held that, over the course of a lifetime, a person who speaks multiple languages experiences the internal interaction of those languages, including competition between or among them e.g., [6]. To manage this competition, speakers of more than one language develop executive functions and associated brain structures that differ from that of monolinguals, who themselves face no such cross-language interaction [7,8]. 

Executive function is a term used to refer to the cognitive control processes that are engaged to manage behavior in service of a goal [9,10]. These processes include the ability to plan and direct attention, ignore distracting information in the environment, inhibit habitual responses, be flexible, and switch among multiple tasks [11]. Collectively, these abilities are referred to as executive functions, and have been linked to success throughout life, including academic achievement, socioemotional wellbeing, and occupational success [12,13,14,15]. Indeed, prior research has shown that when speakers of more than one language use one or another of their languages, the mental representations from the unused language(s) remain active and compete with the one currently in use, thus requiring interference resolution of the cross-language competition [16,17,18]. The argument is that this ever-present cross-language competition induces speakers of multiple languages to continuously inhibit associations and mental representations from the task irrelevant language(s) [19]. According to accounts of the bilingual advantage e.g., [20], it is this constant practice with inhibition (i.e., bilingual language control) that enhances domain-general inhibitory control.

The popularity of the notion of such an advantage helped change attitudes towards bilingualism—from one that was generally negative [21] to one that is now overwhelmingly positive [22]. Nonetheless, the increased research activity has resulted in mixed opinions on the extent to which language control relies on the same mechanism as domain-general executive function processes (especially inhibition) [2,20,23]. Thus, whether such language competition confers bilinguals/multilinguals with a corresponding cognitive advantage has itself become controversial for an overview, see [24]. In the last ten years, studies have provided evidence both for e.g., [25,26] and against e.g., [27,28,29,30] the notion that bilingualism confers any sort of advantage. Meta-analyses of the relevant literature likewise have rendered mixed assessments. Some have rendered positive assessments in this domain e.g., [31,32], while others cast doubt e.g., [33,34,35]. Still others serve to underscore publication bias as a key factor underlying findings of a language-related advantage [36,37]; but see [32,38]. 

Critically, this debate is serving to identify oversights in earlier research. For example, researchers have identified variable operationalizations of bilingualism [39,40,41], methodological confounds of various kinds (and corresponding misinterpretations of findings) [19], publication bias that favors significant findings (and relegates null results to the file drawer) [34,37,42]; but see [32,38], and overly strong conclusions that are not, in fact, supported by the data [33]. Many recent findings reveal the importance of factors that vary across individuals—including age, socioeconomic status (assessed as various amalgams of education, earnings, and neighborhood), culture, and, in particular, second language (L2) usage profiles—in modulating the existence of the bilingual advantage e.g., [43,44]. Thus, the coming decade promises to include much more rigorous investigation of how these and other factors interact with cognitive control to influence language processing in bilingual listeners see also [5].

## 2. Multiple Factors within Bilingualism Affect Cognitive Processing 

As outlined in their recent article in *Brain Sciences*, Pot, Keijzer, and de Bot (2018) [45] suggest that the field needs to re-evaluate how bilingual/multilingual listeners are classified, consistent with some of the arguments already identified e.g., [39], and as we detail further below. The need for clear classification criteria for grouping purposes is particularly acute in the context of research evaluating the existence of a language-related advantage. Multilingualism is a complex construct, with individuals varying not only in the number of languages they know and how proficient they are in each, but also in the social contexts in which they use those different languages. Indeed, Pot et al. (2018) [45] tested the idea that one’s sociocultural environment impacts how a speaker of multiple languages switches among the different languages, which in turn influences whether a cognitive processing advantage arises at the individual level. Their study highlights at least four issues currently being discussed in the wider domain of bilingualism research, which we outline in turn.

First, the key motivator behind the approach these researchers pursued was their hunch that inter-individual variability in the sociocultural context is a contributing factor to the lack of consensus over whether speaking more than one language confers a cognitive advantage. This raises the issue of how bilingualism itself is measured. Pot et al. conducted their study across three regions in the northern Netherlands, all with rich histories in dialectal use, and extensive use of multiple languages. Generally, over 90% of Dutch adults are conversant in one other language (usually English), and well over 50% speak a third as well (commonly German) [46]. Historically, there has been widespread dialectal use throughout the country, although in recent years dialects have been on the decline among younger generations [47]. Pot and colleagues acknowledge this complex history, by measuring each individual’s language background and current use in great detail. Their findings bear this complexity out, revealing that participants spoke an average of four languages. Despite this, the total number of languages spoken was not predictive of positive cognitive effects; rather, only the measures that captured the specifics of the participants’ daily language use were predictive of any benefits in cognitive performance among them, in addition to (but to a greater extent than) other factors such as age, income, and education.

Indeed, that sociocultural context influences language use is not surprising. In recent years, researchers in the domain of bilingualism have moved away from basing their analyses entirely at the group level (e.g., monolinguals vs. bilinguals), whereby measures from the separate groups are averaged and compared [3,48]. This change has been guided by an emerging awareness of the inter-individual differences among bilinguals/multilinguals, particularly in their patterns of language learning and use see [41]. Consequently, researchers now approach bilingualism as something that can be measured on a series of continua, along which individuals can be arrayed e.g., [4,49,50]. Just as in the large body of research that interrogates cognitive processes of monolingual individuals e.g., [51], this approach allows a fuller exploration of the different factors that may correlate with different aspects of cognitive processing in speakers of more than one language. 

A second factor to consider is the age of participants. Moving beyond sociocultural factors, Pot et al. (2018) [45] focused their measurements on a large sample of multilingual older adults. This additional factor acknowledges yet another variable often overlooked in this body of research: where in the lifespan a particular speaker is positioned. Age of language(s) acquisition has long been acknowledged as important [52,53]; less recognized was the notion that one’s current age matters too. There is now widespread recognition that current age group membership is relevant to understanding the cognitive processes engaged by and impacts of using more than one language e.g., [3]. One reason for this is that the longer an individual lives, the more nuanced their language attitudes are likely to be. Sociocultural factors change over time, impacting language use. Likewise, changes in sociocultural attitudes towards the different languages accumulate over the lifespan, further shaping how language is used at any given time.

A third issue to consider is which tasks should be used to understanding the impacts of bilingualism/multilingualism on cognition. For example, Pot et al. (2018) [45] assessed their participants using two tasks commonly used in research on executive function: the Flanker task and Wisconsin card sorting task (WCST). These are by now standard tools in executive function research, including that focused on bilingualism. When coupled with the additional questionnaire-based data from each participant—data comparing participants’ performance on these tasks as a function of how and how often they used their different languages from day-to-day—they were able to provide nuanced processing information. 

A fourth factor that stems from the third is that analyses linking language usage measures to experimental task performance require a range of different analytical tools. For example, Pot et al. (2018) [45] conducted multiple linear regression and partial least squares (PLS) regression analyses to determine which combination(s) of individuals’ language-related and other demographic variables best predicted Flanker or WCST performance. Indeed, methods of analysis have emerged as a focal topic in recent discussions of progress in bilingualism research [5]. In this way, researchers are bringing new approaches to bear in making sense of existing data and in generating new data. On a related note, as we discuss further below, one active area of research is developing new continuous measures of bilingualism to effectively capture one’s nuanced bilingual experience in numerical form. These types of continuous measures will ultimately allow researchers to better link individual differences in bilingual experience with modifications in cognitive performance, as well as brain structure and function. 

In short, Pot et al. (2018) [45] is a great example of an approach that combines these issues: nuanced sociocultural characterization of an individual’s language use, developmentally guided lifespan perspective, and mixed measures of the phenomenon of interest. 

## 3. Key Findings 

Overall, the results reported by Pot et al. (2018) [45] reveal that when multilingualism is characterized within a particular group of speakers by so-called “knowledge factors”, including things like the number of known languages, age of acquisition of those languages, and proficiency in them, individual differences in these factors do not predict individual differences in Flanker or WCST performance. However, when an alternative characterization of multilingualism based on “language usage factors” is included in the regression model, individual differences on the Flanker Task (but not WCST) can be predicted. In particular, the critical factor in the Pot et al. study was how intensely participants used their second language (L2) in different social contexts, and how the intensity of this L2 usage interacted with language switching in real-life social contexts. Specifically, the more “intense” an individual’s dual-language usage patterns in day-to-day life, the more similar their response times (RTs) on the congruent and incongruent Flanker trials (i.e., a smaller Flanker effect). This finding suggests that older adults with intense dual-language usage are better able to select task-relevant information and/or inhibit task-irrelevant information. Moreover, using PLS regression, which incorporated additional demographic variables (e.g., age, income, and personality traits), the researchers found that participants’ language usage, together with certain other personality traits (e.g., openness to experiences) and socioeconomic status-related variables (here, based on income and education), predicted individual differences in Flanker scores. Even in the PLS regression, language usage factors were not predictive of WCST performance, a task issue that we will return to below.

Overall, we interpret these findings as underscoring how real-life, day-to-day language usage, beyond different measures of language knowledge, is associated with cognitive advantages in bilingual/multilingual older adults. Thus, future studies on bilingual effects will continue to benefit from characterizing bilingualism/multilingualism in a holistic manner. While it is important to collect information about participants’ language history (including their known languages, age of acquisition of those languages, and proficiency), special attention should also be given to tracking information about how these individuals use their languages in real-world social contexts. 

## 4. A Way Forward in Bilingualism Research

As we have noted, researchers have begun moving away from group comparisons between monolinguals and bilinguals, and instead are leveraging the diverse experiences represented within bilingual and multilingual populations e.g., [4,54] for reviews and commentaries on this issue, see [3,5,41,48,55]. In the sections that follow, we focus on the four issues we identified within the Pot et al. (2018) study [45] that reflect these emerging issues. We outline some trajectories within recent bilingualism research, the pursuit of which we believe will enhance our understanding of the different ways language experience may modulate cognitive processing.

### 4.1. Measuring Sociocultural Components 

Sociocultural components of bilingualism/multilingualism should influence research design. There are factors, now obvious, which have been shown to impact language processing, including the degree of use (e.g., limited, partial, proficient), mastery of reception and production (e.g., receptive bilingual and productive bilingual), sequence of acquisition (e.g., simultaneous or sequential), and degree of balance (e.g., balanced and non-balanced) for an overview, see [56]. An even broader issue is the sociocultural context in which an individual uses the different languages see [57] for a nuanced interrogation of this issue. These factors include things like whether bilingualism is valued in the place of residence, and the specific contexts in which the different languages are used. For example, in an important demonstration of the power of culture, Zhang and colleagues (2013) asked native Chinese speakers learning English as their L2 in the United States to speak in the L2 with an avatar, which the researchers manipulated to have either a Chinese or Caucasian face [58]. In either case, the researchers found that these US-based native Chinese speakers spoke English, their L2, more hesitantly to an avatar with a Chinese face than to a similar avatar with a Caucasian face. These findings highlight the degree to which native language culture—and cues about it—can influence moment-to-moment language production. While the disruption in L2 speech production occurred when the participants were asked to address individuals who shared their dominant language (in this case, Chinese), this was taking place in a behavioral testing lab in New York City. While the lab no doubt cued the dominant language of the culture, New York is a notoriously diverse city. Which aspects of cultural cuing impacted the participants’ speech production? This is very difficult to tease out, the point being that sociocultural factors include cultural assumptions about when and where the language of the region should be used rather than the presumably more comfortable shared language. Factors such as these are all in play in any given situation see another example in [1]; it stands to reason that they affect an individual’s developmental trajectory, linguistically, cognitively, and otherwise.

In addition to sociocultural effects on the dynamics of moment-to-moment language production and processing in speakers with variable levels of competency in a language, the broader sociocultural environment surely impacts which language(s) are used by individuals who can more naturally switch among languages. Indeed, inter-language switching behavior is a fact of multilingual daily life. Notably, the Pot et al. (2018) study [45] was conducted in the northern Netherlands, a region described by the study’s authors as “rich in dialects and languages” [45] p. 5. The language experience of those older adults can be juxtaposed with those of the Zhang et al. (2013) study, whose Chinese participants had been living in the US for a relatively short amount of time (approximately one year) [58]. This contrast highlights issues having to do with the huge variability that individuals experience in mere language exposure; it also highlights matters having to do with sociocultural acceptance of the different language(s). Furthermore, in a highly bilingual/multilingual society or community, interlocutors may be expected to switch languages more often and in a wider range of social situations. Therefore, in line with Pot et al. (2018) [45], it is critical for researchers to measure individuals’ language usage in real-world social contexts and characterize how the various known languages interface at the community level with the dominant language(s) of the region.

### 4.2. The Importance of Taking a Lifespan Perspective

While the Pot et al. (2018) study [45] focused exclusively on multilingual older adults—individuals aged 65 years and older—further research will be needed to determine if similar effects manifest in other age groups. As it stands, it is unclear if the Pot et al. findings would replicate in younger age groups, people for whom bilingual advantage effects tend to be more elusive, as shown in a recent meta-analysis [44] in which multilingualism-related cognitive advantage effects were observed to be stronger in older than younger adults. Further studies that take a holistic approach to the issue of bilingualism/multilingualism, as exemplified by the Pot et al. approach, will be needed to determine if a finer-grained characterization of language usage reveals comparable results in bilingual young adults and children. 

Focusing on interactions among aging, bilingualism, and cognition, previous studies have demonstrated that speakers of multiple languages have brains that are more resistant than monolingual brains to age-related cognitive decline e.g., [59,60,61]. Evidence has accrued that bilingualism benefits cognitive processing as people age; the argument has been aided further by better control of confounding variables associated with speaking more than one language (e.g., immigration history, overall intelligence). Moreover, better control of age-related factors, including one’s current age and age-of-acquisition of each language, is contributing to a better understanding of the mechanisms that underlie and/or enable age-related cognitive reserve. Notably, this realm of research is revealing the inherent variability among people who speak more than one language. This variability can be attributed to a host of individual and sociocultural factors, highlighting the need for longitudinal research in this domain [5]. Therefore, we applaud researchers’ willingness to embrace this complexity in their study designs, as exemplified by the Pot et al. (2018) study [45]. Greater incorporation of a lifespan perspective in this research will help us better understand the role that age plays in the cognitive processes of bilinguals/multilinguals and monolinguals alike for similar arguments, see [3].

In arguing that the Pot et al. (2018) study [45] takes a lifespan perspective, we wish to highlight the importance of considering an individual’s age—anywhere from infancy to late adulthood—when examining how bilingualism affects language acquisition and cognitive development. As previously mentioned, the view that bilinguals could profit cognitively from their bilingualism is based on the theoretical assumption that bilingual and multilingual individuals experience constant cross-language interaction and competition during language processing [16,17,18]. To use the correct language in any given situation, a cognitive control mechanism must be engaged, allowing speakers to resolve the conflict between actively competing languages. Crucially, this cognitive control mechanism comprises various executive functions that begin to develop in early childhood [11]. Thus, one can imagine that the age at which this developmental process was engaged is critical to impacts observed even years later. But developmental processes do not take place in a sociocultural vacuum. The degree of necessary control can be a product both of internal cognition (inter-language competition), and of sociocultural pressures and assumptions see [1]. Thus, understanding the relationship between these early developmental processes and subsequent cognitive abilities will require both cross-sectional and longitudinal studies that interrogate processing abilities from early childhood to late in life.

### 4.3. Task-Related Considerations

Pot et al.’s (2018) [45] results showed a distinction between the Flanker task and WCST. Specifically, the researchers observed language-usage effects on the Flanker task, but not on the WCST. As they describe in their paper, this pattern of results seems to indicate that intense language usage influences selective attention and inhibition in particular, but not other aspects of executive function. However, these incongruent results on the WCST and Flanker are not surprising. Prior studies have demonstrated that executive function tasks that, at least on the surface seem like they should rely on the same executive function mechanism, do not necessarily correlate e.g., [10,30,62]. Furthermore, the WCST is a complex task, likely tapping into multiple executive processes, such as inhibition of the preceding sorting rule; consequently, care should be taken when inferring the executive function process that contributes to the incongruent Flanker and WCST results for a similar discussion, see [8]. 

Notably, as Valian (2015) describes, various life experiences (not just bilingualism, but also activities like exercise and education) can enhance executive function—a construct in and of itself that is not well-defined [8]. Thus, similar to Valian’s (2015) proposal [8], we believe that a two-pronged approach is needed to disentangle the complicated relationship between bilingualism and executive function: (1) A focus on experimental task design to more precisely characterize and isolate the individual component processes within the executive function umbrella. (2) A focus on individual differences in language usage as well as other demographic variables, within bilingual samples (like the approach of Pot et al. (2018) [45])—to isolate the specific aspect(s) of the bilingual experience that interact to modulate a specific executive or cognitive process for a detailed discussion of this, see [41]. 

There have also been calls to develop more ecologically-valid tasks to assess bilingual effects on executive function components and examine processes—including those outside of the traditional executive function realm—that are relevant for daily life e.g., [2,5]. For example, language switching tasks are commonly used to examine language control processing in bilingual and multilingual individuals [for a review, see 19]. This is an important research avenue, given the proposed relationship between language control and domain-general executive control in bilinguals [20]. However, a recent study by Jyllkä et al. (2020b) directly examined the bilinguals’ frequency of language switching in real life and measured their performance on both executive function tasks (including Simon and flanker tasks) and a cued language switching (picture naming) task [62]. Overall, they found that bilinguals who tended to code-switch more frequently in real life tended to perform worse on the cued switching and executive function tasks [62]. One possible explanation for this unexpected result may be due to the unnatural nature of lab-based tasks. Focusing on language switching tasks, Blanco-Ellorieta and Pylkkänen (2018) postulate that when bilinguals converse in a dense code-switching context (i.e., when interlocutors share the same languages), language switching can occur in a voluntary, unforced manner. This contrasts with lab-based cued naming tasks, which involves a forced language switch [2]. Consequently, these findings and perspectives generate more complex questions about the relationship between real life language switching, the sociocultural landscapes in which languages are used, and executive function also see [6,63]. Only with more ecologically-valid tasks and careful attention to individual differences in language usage, sociocultural landscapes, and other factors, can the field disentangle these complex interactions between the bilingual experience and real-life cognitive processing. 

### 4.4. Other Measurement Tools and Techniques 

While the shift from group-based comparisons to individual differences approaches has already begun to propel the field forward, a lingering issue is how bilingualism should be operationalized. Indeed, this lack of homogeneity across studies, in terms of how bilingualism is defined, measured, and reported, hinders the comparison of results across studies [39,40,41]. Of course, many aspects of the bilingual experience are very qualitative in nature, making them difficult to quantify.

Recently, researchers have begun proposing innovative approaches that could potentially solve this issue, enabling standardized measures that capture various aspects of an individual’s bilingual experience. For example, Marian and Hayakawa (2020) proposed the idea of computing a bilingual quotient (BQ) to capture the intensity of the bilingual experience in a single number, similar to the idea behind IQ [50]; however, the jury is still out in terms of the best way to compute BQ. In a similar vein, Gullifer and Titone (2019) developed a measure of language entropy, which provides a continuous measure of how bilinguals use language in various communicative contexts [49]. Other researchers have developed self-report tools to score bilingual attributes (e.g., language proficiency and dominance) using an updated online version of the language history questionnaire (LHQ3) [64] and to measure language switching in real life using a cell phone app called the Ecological Momentary Assessment (EMA) [65]. These types of measures that capture more fine-grained, meaningful individual differences in the bilingual experience in a continuous and standardized manner should be a focus of future research. Only then can we truly understand which dimensions of the bilingual experience drive neuroplastic changes in brain structure and function and modulate language and cognitive processing. 

Furthermore, as noted by Pot et al. (2018) [45] and others [40,66,67], the missing link in the bilingual effects literature is the explanatory neural mechanism whereby language usage patterns, lifestyle, and/or other relevant environmental factors could enhance specific executive function components in some (but not all) multilingual individuals. Of course, this is a complex and challenging problem, and much more research will be needed to disentangle the role of language usage, lifestyle, and environmental variables on executive function in bilingual and multilingual individuals.

That said, it is important to note that the field is moving in this direction. Several models have been proposed linking various aspects of the bilingual experience with structural and functional neuroplasticity. These models include the Conditional Routing Model [68], the Adaptive Control Hypothesis [69], the Bilingual Anterior to Posterior and Subcortical Shift Model [70], and the Dynamic Restructuring Model [55,71]. Recently, DeLuca et al. (in press) have proposed a theoretical framework called Unifying the Bilingual Experiences Trajectories (UBET) that integrates and builds upon the predictions of the four aforementioned models [72]. Specifically, the UBET framework makes predictions about how different facets of the bilingual experience, including language switching, language usage intensity, and language proficiency, can result in modifications in brain structure, brain function, and cognitive measures. Moreover, UBET’s predictions span both neurophysiological (e.g., electroencephalography (EEG)) and hemodynamic (e.g., fMRI) measures of brain function. Testable frameworks like UBET, which integrate predictions across not only a wide range of bilingual experience factors but also across a variety of outcome measures, are essential to fully map the mechanistic relationships among individual bilingual experiences, neuroplasticity, and cognition.

## 5. Conclusions

To conclude, the coming years in bilingualism/multilingualism research are promising. As observed by Pot et al. (2018) [45], a bilingual advantage in one subset of multilingual individuals does not guarantee such an advantage to all bilinguals/multilinguals. Rather—and as observed across the group of older adults tested in the Pot et al. study—the bilingual advantage is driven at least in part by how individuals use their languages in everyday life. The surrounding sociocultural environment surely contributes to these individual language patterns. Given the complexity and heterogeneity of the bilingual experience, it will be critical in future research to pursue detailed examination of multiple factors—including language usage patterns and sociocultural contexts—via precise tasks, longitudinal approaches, robust measurement tools, and theoretical frameworks that make specific mechanistic predictions about the bilingual experience, neuroplasticity, and cognitive adaptations. Much remains to be understood about how bilingualism and other aspects of cognition interact throughout development, but the future is bright.
